# Molecular Targets for Gastric Cancer Treatment and Future Perspectives from a Clinical and Translational Point of View

**DOI:** 10.3390/cancers13205216

**Published:** 2021-10-18

**Authors:** Justus Körfer, Florian Lordick, Ulrich T. Hacker

**Affiliations:** Department of Oncology, Gastroenterology, Hepatology, Pulmonology and Infectious Diseases, University Cancer Center Leipzig (UCCL), Leipzig University Medical Center, 04103 Leipzig, Germany; florian.lordick@medizin.uni-leipzig.de

**Keywords:** gastric cancer, gastroesophageal cancer, targeted therapy, immunotherapy, personalized therapy, molecular subtypes

## Abstract

**Simple Summary:**

During the last several years, gastric cancer has been extensively studied on the molecular level and distinct molecular subtypes have been defined accordingly. Here, we review the current data on targeted treatment approaches for advanced or metastatic gastric cancer based on molecular alterations or immunological features, ranging from clinically approved treatment strategies to more innovative concepts which are currently in preclinical or clinical development. Information on the translational context and methodology is also provided, holding promise for the further improved development of personalized treatment strategies in the future.

**Abstract:**

Gastric cancer is a leading cause of cancer death worldwide. Systemic treatment comprising chemotherapy and targeted therapy is the standard of care in advanced/metastatic gastric cancer. Comprehensive molecular characterization of gastric adenocarcinomas by the TCGA Consortium and ACRG has resulted in the definition of distinct molecular subtypes. These efforts have in parallel built a basis for the development of novel molecularly stratified treatment approaches. Based on this molecular characterization, an increasing number of specific genomic alterations can potentially serve as treatment targets. Consequently, the development of promising compounds is ongoing. In this review, key molecular alterations in gastric and gastroesophageal junction cancers will be addressed. Finally, the current status of the translation of targeted therapy towards clinical applications will be reviewed.

## 1. Introduction

Gastric cancer is one of the most common tumor entities worldwide with 1 million new cases per year and about 748,000 deaths per year [1]. Systemic treatment is recommended for patients with stage IV gastric or gastroesophageal junction (GEJ) adenocarcinomas. This is based on a broad body of evidence from clinical trials, demonstrating both prolonged survival and control of cancer-related symptoms, translating into improved quality of life [2]. Chemotherapy (i.e., mono- or polychemotherapy) represents an important backbone for systemic therapy. Trastuzumab, an anti-HER2 (human epidermal growth factor receptor 2) antibody, is used in combination with chemotherapy in the first-line treatment of HER2 positive gastric cancer [3]. Ramucirumab, an anti-VEGFR2 (vascular endothelial growth factor receptor 2) antibody, which targets angiogenesis, is approved for second-line treatment either as a monotherapy or in combination with Paclitaxel [4,5,6]. Recently, novel targets like programmed death 1 ligand-1/programmed cell death protein 1 (PD-L1/PD-1), fibroblast growth factor receptor 2 (FGFR2), and claudin 18.2 (CLDN18.2) are emerging. There is urgent need for advances, as prognosis with chemotherapy alone remains poor, with a median survival of about 12 months in metastatic disease.

**Figure 1:**
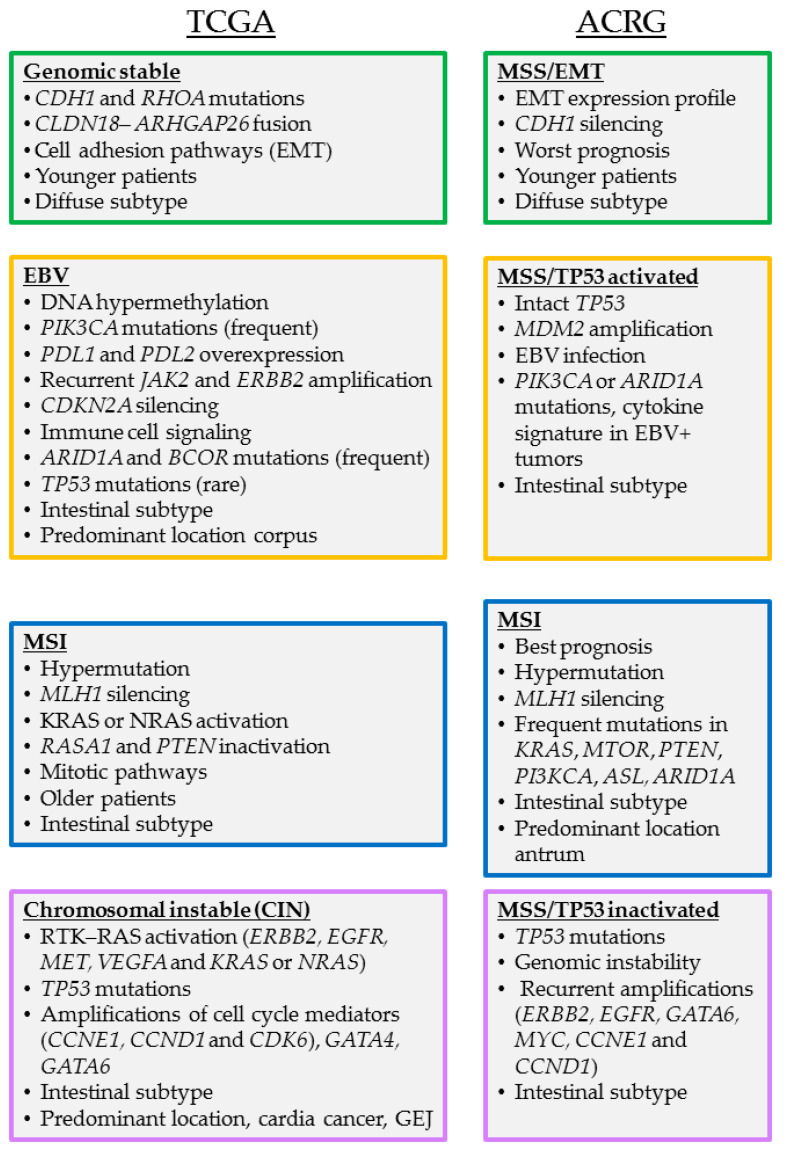
Molecular classification of gastric cancer into distinct subtypes according to TCGA and ACRG.

Morphological and genetic heterogeneity are major hallmarks of gastric cancer and basic histological classifications like the Laurén classification (intestinal, diffuse and mixed subtype) do not allow precise patient stratification towards distinct treatment strategies. From this background, groups in Asia and the US have aimed to more comprehensively characterize the molecular features of gastric cancer. To this end, The Cancer Genome Atlas (TCGA) consortium has performed a comprehensive molecular analysis and classification of gastric adenocarcinomas from 295 patients based on next generation sequencing (NGS), whole-exomes DNA sequencing, mRNA, and miRNA sequencing. In addition, array-based analysis of DNA methylation and reverse-phase protein arrays have been performed. On this basis, four molecular subtypes have been defined: (i) EBV positive (EBV); (ii) microsatellite instable (MSI); (iii) genomically stable (GS) and iv) chromosomal instable (CIN) [7]. In addition, the Asian Cancer Research Group (ACRG) followed a different approach based primarily on gene expression profiling in a cohort of 300 Asian gastric cancer patients to identify four molecular subtypes: (i) microsatellite instable; (ii) microsatellite stable (MSS) TP53 inactive; (iii) MSS TP53 active, and iv) MSS with a gene expression profile related to Epidermal to Mesenchymal Transition (EMT). Furthermore, targeted sequencing and genome-wide copy number microarrays were used to describe key molecular alterations in the four subtypes [8]. The overlap between these two major classifications and key clinical and molecular features are summarized in Figure 1.

Due to differences within patient cohorts studied by these two consortia and due to different techniques used to analyze and define molecular subtypes, overlap is only partial. Specifically, while the TCGA approach relied on several technical platforms, as indicated above, the key technology used by the ACRG was gene expression profiling. There were differences between the cohorts regarding ethnicity and stage distribution (i.e., stage IV disease in 15% of patients in the TCGA data set vs. 26% for the ACRG data set). On the other hand, both classifications were demonstrated to be prognostic. In the TCGA approach ≥ 60% of tumor cell nuclei and ≤ 20% necrosis were required for inclusion, and the ACRG required a similar content of tumor cells. Thus, stroma-rich tumors and aspects related to the tumor microenvironment might be underestimated in both cohorts. Moreover, in both cohorts primary tumor material but not metastases were analyzed, causing another potential bias.

In this review, key molecular alterations in gastric cancer will be addressed and the current status of the translation of targeted therapy towards clinical applications will be comprehensively reviewed. Selected pivotal clinical trials are summarized in Table 1. Furthermore, promising targets under investigation in preclinical trials will be discussed.

## 2. Established Molecular Targets in Gastric Cancer

### 2.1. HER2

Human epidermal growth factor receptor 2 (HER2; syn. ERBB2) has been identified as a key molecular target for gastric cancer treatment even since before the era of comprehensive molecular characterization of gastric cancer began. HER2 belongs to the epidermal growth factor receptor (EGFR) family of tyrosine kinase receptors, which play a pivotal role in carcinogenesis as well as in propagation of tumor cell growth and survival [16,17]. About 20% of patients with gastric or GEJ cancers show an overexpression of the HER2 protein or *HER2* gene amplification [18]. HER2 positivity (HER2+) is defined by either positive immunohistochemistry (IHC) score 3+ or by IHC score 2+ plus fluorescence in situ hybridization (FISH) positivity. No difference in frequencies were found between European and Asian patients, but there were significant differences according to the histological subtype (i.e., 31.8% in intestinal type vs. 6.1% in diffuse type) and location (i.e., 32.2% in GEJ vs. 21.4% in gastric tumors) in the screening cohort of the pivotal ToGA trial [19]. According to the TCGA data set, HER2 positivity rates in the different subtypes were: CIN 24%, EBV 12%, GS 7% and MSI 5% [7]. 

Targeting HER2 is a well-established clinical approach in metastatic HER2+ gastric and GEJ cancers. Several pharmacological approaches have been applied so far to target HER2 in gastric cancer, comprising monoclonal antibodies, antibody-drug conjugates and tyrosine kinase inhibitors (TKI). The pivotal ToGA trial was the first to show improved overall survival (OS) in patients with HER2+ metastatic gastric cancer by adding the anti-HER2 monoclonal antibody Trastuzumab to chemotherapy compared to chemotherapy alone (median OS; 13.8 vs 11.1 months; HR 0.74, 95% CI 0.60–0.91; *p* = 0.0046), and a subgroup with high HER2 expression (IHC score 2+ plus FISH+ or IHC score 3+) benefited most (median OS: 16.0 vs. 11.8 months; HR 0.65, 95% CI 0.51–0.83; *p* = 0.0368) [3]. In contrast, the TKI Lapatinib targeting both EGFR and HER2 did not improve clinical outcome in phase III studies for stage IV HER2+ gastric cancer patients in combination with standard chemotherapy compared to chemotherapy alone, in either first-line [20] or second-line [21] therapy. Moreover, addition of the HER2 targeting antibody Pertuzumab to Trastuzumab did not further improve overall survival in HER2+ metastatic disease in first-line [22]. Finally, the antibody drug conjugate Trastuzumab Emtansin was used in second-line following anti-HER2 therapy in the GATSBY study. Compared to standard taxan treatment there were no statistically significant improvements in clinical outcomes [23]. 

Trastuzumab Deruxtecan is a novel antibody–drug conjugate composed of an anti-HER2 antibody, a cleavable tetrapeptide-based linker and a cytotoxic topoisomerase I inhibitor. Mechanistically, it was shown that even HER2 negative tumor cells are killed due to bystander effects [24]. In the phase II DESTINY-Gastric01 study Trastuzumab Deruxtecan was compared with standard chemotherapy in HER2+ advanced gastric cancer patients having received at least two previous lines of therapy, including anti-HER2 treatment. Objective response rate (ORR; 51% vs. 14%, *p* < 0.001) and overall survival (median OS; 12.5 vs. 8.4 months; HR 0.59; 95% CI, 0.39 to 0.88; *p* = 0.01) were significantly improved compared to standard chemotherapy. Treatment related adverse events were higher in the experimental arm; however, most adverse events were manageable [9]. Therefore, Trastuzumab Deruxtecan was approved by the FDA for patients with advanced HER2+ gastric cancer, who have received a prior Trastuzumab-based regimen. Further studies investigating Trastuzumab Deruxtecan as a monotherapy (DESTINY-Gastric02 (NCT04014075, phase II, western study population), DESTINY-Gastric04 (NCT04704934, phase III)) or in combination with chemotherapy or immunotherapy (DESTINY-Gastric03 (NCT04379596, phase I/II)) are ongoing.

A number of mechanisms leading to resistance against HER2 targeted therapies have been identified. Preclinical models have been used for the analysis of resistance mechanisms for HER2 targeting. Shin et al. established gastric cancer HER2+ patient-derived xenografts (PDX, *n* = 5) which were treated with Trastuzumab, Pertuzumab and a novel HER2 directed antibody (1A12) as a monotherapy or dual blockade. Data demonstrated that different HER2 directed approaches can be analyzed in PDX, and that individual response evaluation can be obtained in this model [25]. Furthermore, a number of mechanisms promoting Trastuzumab resistance have been addressed preclinically, using different cell line models. On this basis, novel treatment approaches can further be validated in PDXs and finally move to early phase clinical trials [26,27,28,29,30,31,32].

From the clinical point of view, a key problem for successfully applying molecular targeted therapies in gastric cancer is intratumoral heterogeneity. With respect to HER2+ gastric cancer, significant inter-observer variability in HER2 assessment could be identified in the recently published VARIANZ study, especially in tumor specimens with an intermediate level of HER2 expression. Importantly, in this study, borderline HER2 positivity and heterogeneity of HER2 expression were proposed as resistance factors for HER2-targeting treatment [18]. Other mechanisms comprise loss of HER2 expression during Trastuzumab treatment [33], alterations in HER2 downstream signaling related to the RAS–phosphatidylinositol 3-kinase (PI3K) signaling pathway, and the activation of bypass pathways. For example, in the CIN subtype, amplification in other receptor tyrosine kinase pathways can be frequently found, including EGFR and MET. Such co-amplifications have been found to mediate resistance towards blockade of single pathways [34]. Moreover, amplification of *CCNE1* coding for the cell cycle regulator cyclin E1 occurs in conjunction with *HER2* amplification and can mediate resistance towards HER2 targeting therapy [35]. More generally, it has been demonstrated that HER2+ GEJ cancers harbor several such preexisting genomic alterations, which confer resistance, but which on the other hand would in principle be targetable [28]. Consequently, combined approaches to target the different pathways altered are promising in the CIN subtype. Comprehensive molecular profiling, however, represents an important prerequisite in order to identify respective alterations or pathway activations.

Furthermore, there are a number of developments which are promising to further improve HER2 directed treatment approaches. As immunotherapy based on the use of immune checkpoint inhibitors is successfully entering the field of gastric cancer treatment (see paragraph “Immune checkpoint inhibition”), combinations of HER2-targeted therapy with immunotherapy are on the horizon. Combination of HER2-targeted therapy with immunotherapy in HER2+ cancers is supported by a number of preclinical studies. Specifically, it has been shown that anti-HER2 treatment with Trastuzumab can lead to upregulation of PD-L1 [36], enhanced dendritic and T cell invasion, induction of HER2 specific T cell responses [37,38], and the initiation of innate immune response by antibody dependent cell-mediated cytotoxicity (ADCC) [39]. In a single arm phase II trial published by Janjigian et al., addition of Trastuzumab to Pembrolizumab in HER2+ metastatic gastric cancer patients (*n* = 37 patients) resulted in an ORR of 91% (95% CI 78–97%) with a median progression free survival (PFS) of 13 months (95% CI 8, 6-NA) and an OS of 27, 3 months (95% CI 18, 8-NA) [40]. This encouraging data resulted in an ongoing phase III trial (KEYNOTE-811) comparing chemotherapy with Trastuzumab plus Pembrolizumab or placebo. Interim analysis revealed a significant increase in ORR in the experimental arm, with durable responses and manageable adverse events [10]. This led to approval of the combination of chemotherapy plus Trastuzumab in combination with Pembrolizumab for previously untreated HER2+ advanced/metastatic gastric cancer by the Food and Drug Administration (FDA).

A novel anti-HER2 monoclonal antibody, Margetuximab, was combined with Pembrolizumab in a single arm phase Ib/II trial in patients with previously treated HER2+ adenocarcinoma of the gastroesophageal junction and stomach (*n* = 95 patients). Margetuximab was designed with an optimized Fc fragment to potentiate innate immune response through ADCC. This combination showed acceptable safety and tolerability. The ORR was 18% (95% CI 11.15–27.93). However, higher objective response rates were seen in HER2+ and PD-L1+ patients [41]. These results led to enforcement of a phase II/III clinical trial investigating Margetuximab in combination with other immunotherapeutic agents [42].

Other novel compounds in clinical development comprise ZW25 (Zanidatamab), a bispecific antibody directed toward two different HER2 epitopes representing the binding sites of Trastuzumab and Pertuzumab, which has shown promising efficacy in preclinical models of different HER2+ cancers [43]. Also, several pan-HER TKIs like Tucatinib showing already efficacy in extensively pretreated HER2+ breast cancer patients and has received market authorization for this indication in combination with Trastuzumab and Capecitabine [44] or Neratinib, which was investigated in in vitro experiments using gastric cancer cell lines [45].

### 2.2. Targeting Angiogenesis

Tumor angiogenesis is a prerequisite of neoplastic growth. Consequently, angiogenesis was identified as a target for cancer therapy decades ago [46,47,48,49,50]. One of the key regulators of tumor angiogenesis is Vascular Endothelial Growth Factor (VEGF). VEGF mediated angiogenesis can be targeted by the anti-VEGF antibody Bevacizumab, the anti-VEGF Receptor 2 (VEGFR2) directed antibody Ramucirumab, and a large number of TKIs targeting either different VEGF receptors (i.e., VEGFR1-3) alone or in combination with other pathways.

In gastric cancer, however, results from the first-line AVAGAST [51] and AVATAR study [52] investigating the combination of Bevacizumab with chemotherapy compared to chemotherapy alone did not show an improvement in overall survival. Interestingly, in AVAGAST, regional differences in efficacy were seen with patients from European and Pan-American regions benefiting more than those from Asia. The reasons for this finding, however, are still unclear. In contrast, the anti-VEGFR2 directed antibody Ramucirumab showed efficacy in second-line treatment either as a monotherapy (REGARD) [4] or in combination with Paclitaxel (RAINBOW) [5]. Moreover, the TKI Fruquintinib (i.e., targeting VEGFR1-3) was effective in combination with paclitaxel (i.e., disease control rate 68%) in heavily pretreated gastric cancer patients in a phase I/II study in Asian patients [53] and a phase III trial in second line with this combination is currently ongoing in Asia (NCT03223376). Another TKI, Apatinib, showed improved OS, again in heavily pretreated Chinese gastric cancer patients, compared to best supportive care [54]. These data, however, could not be confirmed in another international study [55]. Furthermore, all efforts to identify biomarkers predicting efficacy of antiangiogenic treatment in gastric cancer patients thus far have failed [56,57,58,59].

Notably, as no molecular stratification was possible at the time these trials were conducted, in view of the TCGA data set recurrent amplification of the gene encoding for VEGF has been found predominantly in the CIN subgroup of gastric cancer. From this background, it might be hypothesized that this respective subgroup might benefit most from antiangiogenic treatment in general.

### 2.3. Immune Checkpoint Inhibition

Nivolumab monotherapy improved OS compared to placebo in third- or later-line treatment of gastric or GEJ cancer patients from Asia (Japan, South Korea, Taiwan) in the ATTRACTION 2 study, leading to approval in these countries [12]. In a global Phase II study (KEYNOTE-059), pretreated gastric or GEJ cancer patients (cohort I of the trial, n = 259) were randomly assigned to receive Pembrolizumab or placebo as a third- or later-line therapy. The objective response rate was 11.6%. The PD-L1 positive cohort (according to the combined positive score, CPS) showed better objective response rates compared to the PD-L1 negative cohort (15.5% vs. 6.4%) [60]. Based on these data, Pembrolizumab was approved by the FDA for PD-L1 CPS ≥ 1 gastric and GEJ cancer patients after disease progression (i.e., at least two previous treatment lines). In contrast, the second-line study KEYNOTE-061 addressing the role of immune checkpoint monotherapy (i.e., Pembrolizumab) vs. standard of care chemotherapy did not show a survival benefit [61]. Furthermore, the JAVELIN gastric 100 study investigating immune checkpoint blockade with Avelumab as a maintenance strategy following induction chemotherapy was also negative [62]. Tumor mutational burden (TMB) was studied in the KEYNOTE-061 study as a biomarker beyond PD-L1 expression and predicted efficiency of Pembrolizumab treatment [61,63] even when patients with MSI were excluded [63]. Specifically, a TMB beyond the cut-off value of 175 mutations/exome was significantly associated with ORR, PFS and OS in patients treated with pembrolizumab [61]. Similar results were obtained with the alternative Foundation One® CDx test using a cut-off of 10 mutations/megabase [63].

Recent data on the addition of immune checkpoint blockade to chemotherapy in locally advanced irresectable or metastatic GEJ or gastric cancer in the first-line setting were in part contradictory. In the KEYNOTE-062 phase III study, Pembrolizumab monotherapy was non-inferior to chemotherapy [11]. However, Pembrolizumab mono or Pembrolizumab plus chemotherapy were not superior to chemotherapy alone regarding OS and PFS endpoints. In the Asian ATTRACTION 4 study, PFS was improved with the addition of Nivolumab to chemotherapy in first-line therapy of gastric or GEJ cancer patients compared to chemotherapy alone. However, no improvement in OS was found and PD-L1 (tumor positive score) did not identify a subgroup with improved outcomes [64,65]. TMB was studied in both trials on an exploratory basis as an additional biomarker for immune checkpoint therapy [13,66], indicating that there might be a predictive role. On the other hand, the CheckMate-649 study demonstrated a significant improvement both for OS and PFS when adding Nivolumab to chemotherapy versus chemotherapy alone in previously untreated patients with advanced gastric or GEJ cancer with a prespecified PD-L1 expression CPS ≥ 5 [67]. In the same direction, not focusing on gastric cancer patients, the KEYNOTE-590 demonstrated that addition of Pembrolizumab to chemotherapy in first-line treatment improved OS and PFS in GEJ adenocarcinoma patients with a PD-L1 CPS ≥ 10 compared to chemotherapy alone [68].

Regarding the TCGA classification, MSI and EBV subtypes have been shown to benefit extensively from immune checkpoint blockade [69]. High TMB resulting in extensive formation of cancer-specific neoantigens triggering robust anti-cancer immune responses is regarded as a key driver in the MSI subtype. Within the EBV subtype of gastric cancer, PD-L1 overexpression was shown to be triggered by two mechanisms: focal amplification of *CD274* (PD-L1) and IFN-γ-mediated signaling via activation of IRF3 to increase PD-L1 expression [70]. Interestingly, 9p amplifications are enriched in the EBV subgroup (15% of tumors) according to the TCGA data set [7]. The genes *CD274* and *PDCD1LG2* are located in this region, and encode PD-L1 and PD-L2. Moreover, *JAK2* amplification, also often present in the EBV gastric cancer subtype, may even enhance PD-L1 expression, as has been shown in the context of triple negative breast cancer. Remarkably, in classical Hodgkin´s Lymphoma (cHL), 9p amplifications are a defining feature [71]. Of note, cHL is extremely sensitive to immune checkpoint blockade [72].

Accordingly, in a prospective phase II study, an ORR of 100% was reported for EBV positive gastric cancer patients, and ORR was even slightly lower (i.e., 85.7%) in MSI gastric cancer patients [69]. Analysis of concurrent alterations in EBV positive gastric cancer (i.e., *PIK3CA* mutations, *HER2* amplification) appear promising to identify mechanisms of resistance towards immunotherapy or to propose novel combinations [73]. Combinations of multi-TKI with immune checkpoint inhibitors are currently in clinical development. Promising data from a phase II trial exist for a combination of Regorafenib with Nivolumab in a cohort of microsatellite stable (MSS) mismatch repair proficient colorectal cancer patients [74], and in the Phase Ib REGONIVO, EPOC1603 trial toxicities were manageable in colorectal and gastric cancer patients. The gastric cancer cohort comprised 25 patients treated with ≥ 2 previous treatment lines. ORR of 44% and a median PFD of 5.6 months were promising [75]. Moreover, the multi TKI Lenvatinib was tested in combination with Pembrolizumab [76] in the phase II LEAP-005 study, and a phase III trial in patients with advanced or metastatic gastric or GEJ cancer in combination with chemotherapy in the first-line treatment is underway (NCT04662710).

## 3. Potential Novel Molecular Targets for Gastric Cancer Treatment

### 3.1. EGFR

The epidermal growth factor receptor (EGFR; syn. ERBB1) has been extensively studied as a target in gastric cancer based on preclinical findings that this pathway is involved in tumor progression and metastasis [17]. While overexpression of EGFR at the protein level was reported at variable frequencies ranging from 30–60% in diverse cohorts [16], *EGFR* amplification was found in only 4% of cases from the TCGA data set and pathway activation was reported in 21%, with a predominance in the CIN subtype (10%). Initial unselected phase III trials adding EGFR antibodies (Cetuximab or Panitumumab) to standard chemotherapy did not find improvement in clinical outcomes of patients with advanced or metastatic gastric or GEJ cancer in the first-line setting in the EXPAND or REAL3 trial, respectively [77,78]. Notably, in the REAL3 trial the OS outcome for the combination of EOX (i.e., Epirubicin, Oxaliplatin, Capecitabine) with Panitumumab was significantly worse compared with the standard arm of EOX [78]. Moreover, a study using the TKI Gefitinib in second to fourth line treatment did not demonstrate improved outcomes compared to standard chemotherapy [79]. Biomarker analysis from the EXPAND trial indicated that a very small subgroup of patients with very high EGFR expression (i.e., measured by IHC) might have a benefit from the addition of Cetuximab, while data on the amplification status were not available [80]. In a large cohort of gastric cancer patients from the U.S., *EGFR* amplification frequency was 6% and the retrospective cohort and patients in the prospective part of the study (*n* = 140; *n* = 8 amplified), treated at different treatment lines, showed a benefit for EGFR-directed therapy (i.e., Cetuximab or ABT-806, an investigational anti-EGFR antibody) according to the primary endpoint best overall response: complete response 3/7 (43%), partial response 1/7 (14%) and disease control 3/7 (43%) [79]. Importantly, alterations in related pathways as well as tumor heterogeneity were identified as resistance mechanisms (loss of *EGFR* amplification, *PTEN* deletions, *KRAS* amplifications/mutations, *NRAS*, *MYK* and *HER2* amplifications) [81]. Furthermore, ctDNA analysis was able to monitor EGFR amplification status during treatment and correlated with the efficacy of anti-EGFR treatment. Finally, increased immune cell infiltration (i.e., CD3+ T-cells, NK-cells) as well as increased PD-L1 expression, which is otherwise low in EGFR amplified patients, were observed in on-treatment biopsies but were lost at progression. These findings, relying on small numbers, are hypothesis-generating, suggesting combinations of anti-EGFR antibodies with immune checkpoint inhibition [81]; however, this needs validation in larger cohorts. Recently, data from the REAL3 trial [78] have indicated that *EGFR*-amplified cases can been identified by analysis of circulating tumor DNA (ctDNA) using droplet polymerase chain reaction or in tumor tissue using FISH analysis. Interestingly, in contrast to previous results showing a benefit for anti-EGFR antibody monotherapy in EGFR amplified gastric cancer patients [81], *EGFR*-amplified patients from REAL3 treated with the EGFR antibody Panitumumab plus EOX chemotherapy had a particularly adverse survival outcome compared to chemotherapy alone [82]. Mechanistically, using tumor organoid models, an antagonistic effect between anti-EGFR agents and the chemotherapy drug Epirubicin specifically in *EGFR*-amplified organoids was found [81], indicating, that the chemotherapy backbone might have an impact on the outcome.

### 3.2. MET Alterations

The *MET* gene encodes Hepatocyte Growth Factor (HGF) receptor, which is a member of the receptor tyrosine kinase family involved in the regulation of cell growth and differentiation in health and disease. In gastric cancer, MET represents a major oncogenic driver (i.e., proto-oncogene), and activation of the pathway promotes disease progression [83] as well as resistance to HER2-targeted treatment strategies [84] and represents an adverse prognostic factor [85]. Important growth-promoting downstream pathways are activated by MET, such as the PI3K/AKT/mTOR pathway the RAS/RAF/ERK/MAPK pathway, the STAT-3 pathway and NFkB. Consequently, treatment strategies have been developed to target MET pathway activation. While up to 50% of gastric cancers show an overexpression on the protein level by IHC, only 3–4% are characterized by *MET* gene amplification [86] and this often occurs together with amplifications in *EGFR*, *HER2* and other receptor tyrosine kinases and is related to the CIN subtype according to TCGA [7].

Following promising data from a phase II study in gastric cancer patients overexpressing MET [87], the monoclonal antibody Rilotumumab targeting the MET ligand HGF has been tested in phase III studies in first-line treatment of metastatic gastric cancer patients with MET positive tumors (i.e., according predefined IHC-based criteria), in combination with standard chemotherapy. The international RILOMET-1 study, however, failed to demonstrate an improvement in survival outcomes [88] and the RILOMET-2 trial [89], which was exclusively focused on Asian patients, was terminated prematurely due to an increased risk of death with the study drug Rilotuzumab in an interim safety analysis. Similarly, a phase III trial of the antibody directly targeting MET (Onartuzumab) in metastatic gastric cancer patients with MET positive tumors according predefined IHC-based criteria in combination with standard chemotherapy failed to demonstrate a survival benefit [90]. In addition, several TKIs targeting MET have been studied in gastric cancer patients in phase I or II trials. In the VIKTORY umbrella trial, there was a promising signal (i.e., ORR 50%) for the activity of the MET inhibitor Savolitinib in gastric cancer patients, and this compound is currently under further clinical development [91]. Mostly, however, efficacy of MET targeting TKI was marginal [83]. 

Key factors contributing to the disappointing results thus far are related to the heterogeneity of target expression found in gastric cancer, difficulties in reliably measuring MET overexpression/activation, and the activation of alternative signaling pathways specifically in the TCGA CIN subgroup, characterized by multiple concurrent amplifications in related pathways [83]. Moreover, translational research data from studies related to the VIKTORY trial indicated that novel occurring mutations in MET (i.e., MET D1228V/N/H and Y1230C) or high copy number MET gene amplifications contribute to the development of resistance against Savolitinib, which can be identified in the circulating tumor DNA (ctDNA) from blood samples [92]. Consequently, novel combination approaches targeting such related pathways and combinations with immune checkpoint inhibitors are promising; as for the latter, MET amplification has been identified as a resistance mechanism in lung cancer [93]. Moreover, MET Exon 14 skipping mutations, which leads to an activation of the MET pathway, can be targeted by specific TKIs (i.e., Capmatinib, Tepotinib). These compounds have already received market authorization for the treatment of Non-Smallcell Lung Cancer (NSCLC) patients harboring this alteration based on phase III studies [94,95]. Since MET Exon 14 skipping mutations have also been identified in gastric cancer in up to 7% of cases, these compounds are very promising for this subgroup.

### 3.3. FGFR2 Alterations

Alterations in FGF receptor genes are found in a wide variety of cancer entities ranging from < 1% in sarcomas to 32% in urothelial carcinoma [96]. In gastric and GEJ cancers, frequency ranges between 3–7% and amplifications are the most common alteration, followed by rearrangements and mutations [96]. In a cohort of 269 gastric cancer patients, the overall frequency of *FGFR2* alterations was 4%, consisting of 72% amplifications, 13% mutations and 8.6% rearrangements. Multiple *FGFR2* alterations were found in 6.3%. The level of amplification was shown to negatively affect chemotherapy response and prognosis [97]. FGFR2 pathway activation is overrepresented in the GS (9%) and CIN (8%) subtypes according to TCGA. With regard to biomarker development, *FGFR2* amplification was shown to predict efficacy of the multi TKI Regorafenib, targeting FGFR2 amongst others, in preclinical models [97]. Small molecular compounds targeting FGFR have successfully been introduced in the clinic for the treatment of urothelial cancer (i.e., Erdafitinib, a pan-FGFR inhibitor) or cholangiocarcinoma (i.e., Pemigatinib, targeting FGFR1-3) and companion biomarkers related to FGFR alterations have been developed [98,99]. In gastric cancer, AZD4547, a selective FGFR-1, 2, 3 TKI which showed good preclinical activity in *FGFR2* amplified gastric cancer patient derived xenograft models [100,101], failed to improve PFS in the second-line treatment of advanced gastric cancer compared to paclitaxel; intratumoral heterogeneity of FGFR2 expression was proposed as a possible explanation [102].

Moreover, a novel covalent, irreversible FGFR inhibitor (TAS-120, Frutibatinib) is in clinical development [103]. This TKI overcomes resistance to ATP-competitive FGFR inhibitors [104]. A clinical trial in a mixed cohort of advanced cancer patients including gastric cancer patients harboring *FGFR2* alterations is currently underway [105]. Finally, Bemarituzumab, which represents a first-in-class humanized afucosylated IgG1 monoclonal antibody to FGFR2b (i.e., a splice variant of FGFR2) has shown promising clinical data in a phase I study in advanced solid tumors and FGFR2b selected gastric and GEJ cancer patients [15]. In the pretreated gastric and GEJ cohort characterized by *FGFR2* amplification, a partial response rate of 17.9% was reached [15]. In a phase II study (FIGHT trial) Bemarituzumab plus chemotherapy (i.e., FOLFOX) was tested in first-line therapy in patients with unresectable locally advanced or metastatic HER2 negative gastric cancer with FGFR2b overexpression (i.e., IHC or *FGFR2* amplification by circulating tumor DNA (ctDNA) analysis). Specifically, median PFS was improved to 9.5 months in the Bemarituzumab arm, compared to 7.4 months (hazard ratio (HR), 0.68; 95% confidence interval (CI), 0.44–1.04; *p* = 0.07) in the placebo arm. Median OS was not reached in the Bemarituzumab arm vs. 12.9 months in the placebo arm (HR, 0.58, 95% CI, 0.35–0.95; *p* = 0.03). ORR was improved from 40% to 53% in the Bemarituzumab arm [106,107]. While the treatment was overall well tolerated, eye toxicity (i.e., corneal adverse events) was more common in the Bemaritzumab arm (67% vs 10%) [108], representing a class effect of FRGR-inhibitors.

### 3.4. Claudin 18.2 

The TCGA gastric cancer study has identified CLDN18-ARHGAP26/6 fusions in 15% of the patients, which predominantly occurred in the genomically stable subtype [7]. Nearly all tumors with CLDN18-ARHGAP26/6 fusions show claudin 18.2 (CLDN18.2) expression [109]. CLDN18.2 is an isoform of the claudin protein and belongs to structural components of tight junctions. It is expressed in various types of tumors but not in normal tissue except gastric mucosa [14]. In gastric cancer, about 40% of patients present with an overexpression according to immunohistochemistry [110]. The monoclonal antibody Zolbetuximab binds to CLDN18.2, and induced cancer cell death through antibody-dependent cellular cytotoxicity and complement-dependent cytotoxicity. Zolbetuximab was studied in two phase II trials as a single agent therapy (MONO trial) and in combination with chemotherapy (FAST trial) in advanced CLDN18.2 positive gastric cancer patients [111,112]. The FAST trial showed beneficial effects of adding Zolbetuximab to standard first-line chemotherapy with prolonged PFS (HR 0.44; 95% CI 0.29–0.67; *p* < 0.0005) and OS (HR 0.55; 95% CI 0.39–0.77; *p* < 0.0005) in CLDN18.2 positive (>40% positive tumor cells) gastric or GEJ cancer [111]. Further validation of these results is currently being attempted in two phase III trials (NCT03504397, NCT03653507), which are investigating Zolbetuximab in combination with chemotherapy in the first-line setting in CLDN18.2 positive (>75% positive tumor cells) gastric and GEJ cancer. In addition, CLDN18.2 is being studied as a target for CAR-T cells (NCT04404595, NCT04467853) as well as for a bispecific T cell engager (BiTE) (NCT04260191) in phase I trials. The AMG 910 trial is investigating a CLDN18.2/CD3 directed BiTE to trigger tumor cell lysis by inducing T-cell activation and proliferation in advanced stage gastric and GEJ cancer patients.

### 3.5. MUC17

Another promising target in gastric and GEJ cancers is mucin 17 (MUC17), which belongs to the mucin family of glycosylated protein. Mucins are transmembrane proteins of the mucosal barrier of normal gastrointestinal epithelial cells [113]. MUC17 is overexpressed in gastric cancer cells, and expression in normal cells is limited. Therefore, a BiTE designed to engage MUC17 positive cells and CD3 positive cells is currently being investigated in a phase I trial ([114], NCT04117958).

### 3.6. Matrix Metalloproteinase-9 

In cancer, matrix metalloproteinases are commonly upregulated in the tumor microenvironment. Matrix metalloproteinase-9 (MMP-9) is an extracellular enzyme which is involved in angiogenesis, matrix remodeling, tumor growth and metastases. It has been shown that inhibition of MMP-9 leads to improved penetration of chemotherapeutics and immune cells [115]. In early gastric cancer, MMP-9 expression levels were prognostic for survival [116]. To target MMP-9, the monoclonal antibody Andecaliximab was developed and encouraging results from a phase I trial investigating Andecaliximab in combination with chemotherapy in HER2 negative gastric and EGJ cancer led to initiation of a phase III trial in the first-line setting. Disappointingly, addition of Andecaliximab to chemotherapy did not improve OS in patients with untreated HER2-negative gastric or GEJ adenocarcinoma [117,118].

### 3.7. Homologous Recombination Deficiency

Homologous recombination deficiency (HRD) sensitizes tumor cells towards treatment with PARP inhibitors leading to synthetic lethality. This treatment approach is now well established in several cancer entities [119,120,121,122,123,124], and germline or somatic BRCA mutations represent the most widely used biomarkers. Other mutations are also related to HRD, such as *ATM*, *PALB2*, and *RAD51*. Furthermore, loss of heterozygosity represents another characteristic phenomenon in HRD deficient tumors. Accordingly, different methods have been used to identify HRD tumors. These comprise sequencing approaches to identify mutations in HRD related genes or to identify genomic scars resulting from HDR deficiency, and analysis of chromosomal aberrations as a consequence of HDR. On this basis, efforts are ongoing to establish and validate optimized biomarkers in order to define HDR in the context of treatment with PARP inhibitors [125]. In gastric cancer, the frequency of mutations in HDR related genes (22 genes analyzed) related to HDR was overall 32% in a large cohort (*n* = 395). Amongst them, *ATM* was the most frequently mutated with a frequency ranging from 19% in mucinous adenocarcinoma to 4% in diffuse type adenocarcinoma [126]. Another group has identified a mutation pattern characterized by the occurrence of large (i.e., ≥ 3 base pairs) indels to predict efficacy of Cisplatin treatment in pancreatic cancer even in the absence of *BRCA* mutations; this mutational pattern was found in up to 12% in gastric cancer, mostly in the mixed (9%) and the intestinal type (12%) according to Laurén classification [127]. Consequently, treatment with PARP inhibitors might have a role in a distinct subgroup of gastric cancer patients with HDR deficiency. In the REAL3 study patients were treated with chemotherapy (i.e., EOX) with or without Panitumumab and translational studies indicated that greater loss of heterogeneity was associated with longer OS, possibly attributable to the increased activity of platinum compounds (i.e., Oxaliplatin) in HDR deficient tumors [128]. In a cohort of familial diffuse gastric cancer not mutated in *CDH1*, germline mutations in HDR associated genes (i.e., *BRCA1, PALB2, RAD51C*) were identified in 6/31 patients (6.5%); such patients might also benefit from treatment with cisplatin and/or PARP inhibitors [129].

In the unselected phase II study 39 (NCT01063517), second-line treatment of Asian patients with Olaparib plus Paclitaxel versus placebo plus Paclitaxel showed an improvement in PFS. Moreover, a significant improvement in the secondary endpoint OS was seen in a subgroup of patients with ATM negative (i.e., according to IHC) tumors [130]. Based on these data, the phase III GOLD trial was performed, which failed to demonstrate an OS benefit in a similarly selected cohort of Asian patients [131]. Further translational analyses (i.e., NGS-based analysis of 15 HDR related genes and analysis of loss of heterogeneity) indicated better outcomes in the cohort (22 of 378 patients) with ATM mutations [132]. 

In general, how to best identify HDR is still a very important research area for predicting the efficacy of PARP inhibitors beyond BRCA testing. Furthermore, combinations of PARP inhibitors with immune checkpoint blockade, small molecule inhibitors or anti-angiogenic compounds are under way [133] and initial results are promising [134].

### 3.8. Potential Targets in Genomically Stable Gastric Cancer

The GS subtype of gastric cancer carries the most adverse prognosis. It is characterized by the activation of Epithelial to Mesenchymal Transition (EMT) programs. RHOA mutations and CLDN18-ARHGAP6 or ARHGAP26 fusions were found in this subtype, and the frequency of CDH1 mutations was 26% in the TCGA data set. Functionally, all these factors are related to cell motility and invasion. This corresponds very well with the finding that the GS subtype mostly corresponds to the diffuse histological subtype of the Laurén classification, characterized by a discohesive growth pattern. Currently, it is likely that CLDN18-ARHGAP6 or ARHGAP26 fusions will be targetable with CLDN18.2 antibodies, though still unproven. However, *RHOA* mutations were demonstrated to promote Focal Adhesion Kinase (FAK) activation and dependency [135]. Moreover, FAK representing a cytoplasmic protein tyrosine kinase, is overexpressed in several solid cancers, and the development of small molecule FAK inhibitors makes this pathway druggable [136]. On the other hand, sporadic early- onset diffuse gastric cancer is characterized by a high frequency of somatic *CDH1* alterations, and as ROS-1 inhibition has been demonstrated to confer synthetic lethality in *CDH1* mutated breast cancer [137]; this approach may represent an attractive strategy for the treatment of gastric cancer with *CDH1* alterations as well.

## 4. Approaches to Further Improve Precision Medicine in Gastric Cancer

### 4.1. Molecular Tumor Boards

Despite recent development of established novel targeted therapies in gastric cancer, translation from bench to bedside often fails as most drugs selected by preclinical testing are not effective in phase II/III clinical trials [138]. The availability of high throughput sequencing technologies and of several targeted therapies has resulted in more personalized treatment approaches in late-stage disease. Molecular tumor boards which discuss individual patients’ high throughput sequencing reports in order to suggest individual treatment approaches are commonly available in comprehensive cancer centers. It has been shown that the implementation of molecular tumor boards and individual treatment decision making seem to lead to improved outcomes compared to conventional therapy [139]. However, these technologies are still expensive and not yet available for most patients from a global perspective. Additionally, the ultimate proof of individual treatment decisions for non-approved drugs based on molecular tumor board recommendations is still lacking.

### 4.2. Ex Vivo Tumor Models

Besides the genomic approach, patient-derived ex vivo tumor models have been investigated as an approach for individual patient stratification [140]. Essentially, in these models, tumor specimens are collected and utilized to establish patient derived xenografts, organoids or tissue cultures [141,142,143]. For gastric cancer, several studies of gastric cancer organoids have been performed and predictability of individual response to treatment and patient outcomes was observed [144,145,146]. Furthermore, patient derived tissue cultures have been established for gastric and GEJ cancer [147]. It was also shown that this model can be used to study PD-1 inhibition ex vivo, as immune contexture can be preserved in patient derived tissue cultures [148]. However, due to the complexity of this model, the predictive values for patient response to specific therapies have not yet been proved. Additionally, patient derived xenografts have been investigated by several groups and the potential for response prediction and analysis of targeted therapy for specific gastric cancer subtypes have been performed [149,150,151,152]. Although preclinical tumor models can be used for response prediction, analysis of resistance mechanism, and tumor biology, none have been implemented in daily clinical routine due to missing validation in bigger cohorts, high costs and extensive workload.

### 4.3. Novel Trial Designs

Novel clinical trial designs such as basket and umbrella trials are increasingly used to study the efficacy of biomarker directed targeted therapy strategies. In the area of gastric cancer, the PANGAE and VIKTORY studies are important examples [91,153].

The VIKTORY (targeted agent eValuation In gastric cancer basket KORea) trial aimed to attribute metastatic gastric cancer patients to specific targeted drugs based on eight different biomarker groups identified by NGS (*RAS* aberration, *TP53* mutation, *PIK3CA* mutation/amplification, MET amplification, and MET overexpression all negative, *TSC2* deficient, or *RICTOR* amplification). Based on the results, patients were assigned to 10 different associated clinical trials for second-line treatment with the following drugs: Capivasertib (AKT inhibitor), Savolitinib (MET inhibitor), Selumetinib (MEK inhibitor), Adavosertib (WEE1 inhibitor), and Vistusertib (TORC inhibitor), which were tested with or without chemotherapy. Some 772 patients with gastric cancer were enrolled, and 105 patients finally received biomarker-assigned drug treatment. Compared with standard second-line treatment, the biomarker-assigned treatment cohort had encouraging response rates and survival (mOS 9.8 vs. 6.9 months, median PFS 5.7 vs. 3.8 months) [91]. Moreover, ctDNA analysis demonstrated good correlation between high MET copy numbers and treatment efficacy, indicating that tumor heterogeneity can be captured by this method [91].

In the PANGEA phase II trial [153], a personalized treatment strategy was applied in gastric cancer patients: antibodies targeting HER2, MET, FGFR2 EGFR PD-L1 or VEGFR2 were added to chemotherapy in first- to third-line treatment in gastric or GEJ cancer patients. Treatment selection was done according to a panel of biomarkers (i.e., amplifications in *HER2, MET, FGFR2, EGFR*) or biomarkers related to efficiency of immune checkpoint therapy (i.e., MSI high, EBV positive, PD-L1 CPS ≥ 10 or TMB ≥ 15 mutations/megabase). One-year survival rate (*n* = 68 patients) was 66% and median OS was 15.7 months. Moreover, first-line response rate (74%), disease control rate (99%), and median progression-free survival (8.2 months) were superior to historical controls. Importantly, in case metastatic tissue specimens were not sufficient to complete all assays and biomarker assignment, ctDNA could be used for biomarker assignment, underscoring the potential future role of this method for targeted treatment approaches in gastric cancer. The clinical utility of ctDNA analysis in advanced gastrointestinal cancers has recently been validated [154] and this approach is very promising for assigning gastric cancer patients for targeted therapies in future clinical trials [155].

## 5. Conclusions

Various targeted therapy approaches have been investigated and specific biomarkers like HER2, MSI and PD-L1 currently play a pivotal role in clinical treatment decision making in gastric cancer. HER2 targeted therapy and anti-angiogenic treatment in combination with chemotherapy represent well-established therapeutic strategies in stage IV gastric cancer, and immune checkpoint blockade is rapidly evolving as a therapeutic strategy as well. Several clinical trials investigating targeted therapy in gastric cancer have failed, however, due to tumor heterogeneity and insufficient biomarker-based stratification approaches. Despite this drawback, new molecular targets (e.g., CLDN18.2, FGFR2) have been identified in subgroups of patients, and promising treatment efficacy was observed in several clinical trials. In addition, new treatment modalities like bispecific T-cell engager and CAR-T cells are under investigation. Finally, the TCGA dataset has built a roadmap for the development of novel molecularly stratified treatment approaches. Based on this, specific genomic alterations may serve as novel treatment targets, as they already are or will become druggable by the development of promising compounds in the future.

## Figures and Tables

**Table 1:** Selected pivotal clinical trials in gastric and esophagogastric junction cancers.

Trial	Study Arms	Efficacy Outcomes			Clinical Implications	Ref.
		ORR	mPFS	mOS		
**HER2**						
ToGA *n* = 594 phase III trial, metastatic HER2+ G/GEJ cancers, first line	CTx (Capecitabine or 5- FU plus Cisplatin)	35%	5.5 months	11.1 months	Trastuzumab plus CTx is standard of care in first-line treatment in metastatic HER2+ disease.	[3]
	CTx (Capecitabine or 5- FU plus Cisplatin) with Trastuzumab	47%, *p* = 0.0017	6.7 months, *p* = 0.0002	13.8 months, *p* = 0.0046		
DESTINY-Gastric01 *n* = 188 phase II trial, HER2+ Asian metastatic gastric cancer patients, third or later-line	CTx (Irinotecan or Paclitaxel)	14%	3.5 months	8.4 months	FDA approval for Trastuzumab Deruxtecan in HER2+ G/GEJ cancer patients who have received a prior Trastuzumab-based regimen. No approval in Europe yet.	[9]
	Trastuzumab Deruxtecan	51%, *p* < 0.001	5.6 months, *p* = 0.01	12.5 months		
KEYNOTE-811 *n* = 264 phase III trial, metastatic HER2+ G/GEJ cancers, first-line, interim analysis	CTx (CAPOX or 5FU plus Cisplatin) plus Trastuzumab	51.9%	No data	No data	No mature data yet. Combination of HER2 targeting and immune checkpoint inhibition might have synergistic effects.	[10]
	CTx (CAPOX or 5FU plus Cisplatin) plus Trastuzumab with Pembrolizumab	74.4%, *p* = 0.00006	No data	No data		
**VEGFR**						
REGARD *n* = 355 phase III trial, metastatic G/GEJ cancers, second-line	placebo	3%	1.3 months	3.8 months	Ramucirumab mono therapy is approved for second-line treatment in G/GEJ cancers.	[4]
	Ramucirumab	3%, *p* = 0.76	2.1 months, *p* < 0.0001	5.2 months, *p* = 0.047		
RAINBOW *n* = 665 phase III trial, metastatic G/GEJ cancers, second-line	CTx (Paclitaxel)	16%	2.9 months	7.4 months	Ramucirumab in combination with Paclitaxel therapy is approved for second-line treatment in G/GEJ cancers.	[5]
	CTx (Paclitaxel) with Ramucirumab	28%, *p* = 0.0001	4.4 months, *p* = 0.0001	9.6 months, *p* = 0.017		
**PD-1**						
ATTRACTION-4 *n* = 724 phase II trial, metastatic Asian G/GEJ cancer patients, first-line	CTx (S-1 or Capecitabine plus Oxaliplatin)	47.8%	8.34 months	17.15 months	Biomarker based patient selection is needed. In second-line tretament or later MSI high G/GEJ cancers do benefit from immune checkpoint blockade.	[11]
	CTx (S-1 or Capecitabine plus Oxaliplatin) with Nivolumab	57.5%, *p* = 0.0088	10.45 months, *p* = 0.0007	17.45 months, *p* = 0.26		
ATTRACTION-2 *n* = 493 phase III trial, metastatic Asian G/GEJ cancer patients, third or later-line	placebo	0%	1.45 months	4.14 months		[12]
	Nivolumab	11.2%	1.61 months, *p* < 0.0001	5.26 months, *p* < 0.0001		
CheckMate-649 *n* = 1,581 phase III trial, metastatic patients with oesophageal, gastric or GEJ cancers, first-line	CTx (Capecitabine plus Oxaliplatin or FOLFOX)	45%	6.1 months	CPS ≥ 5: 11.1 months; All patients: 11.6 months	FDA approved Nivolumab in combination with chemotherapy as a first-line therapy in metastatic G/EGJ cancers. EMA decision is still pending.	[13]
	CTx (Capecitabine plus Oxaliplatin or FOLFOX) with Nivolumab	60%, *p* < 0.0001	7.7 months, *p* < 0.0001	CPS ≥ 5: 14.4 months, *p* < 0.0001; All patients: 13.8 months, *p* = 0.0002		
	Nivolumab plus Ipilimumab	No data	No data	No data		Not published yet
**Claudin 18.2**						
FAST *n* = 161 phase II trial, metastatic CLDN18.2+ (≥40% tumor cells) G/GEJ cancers, first-lin	CTx (ECX)	33.3%	5.3 months	8.4 months	CLDN18.2 might be a promising target in future. Further validation is currently ongoing.	[14]
	CTx (ECX) with Zolbetuximab	49.4%, *p* = 0.022	7.5 months, *p* < 0.0005	13.0 months, *p* = 0.0008		
**FGFR2**						
FIGHT *n* = 155 phase II trial, metastatic G/GEJ cancers (FGFR2b overexpression), first-line	CTx (modified FOLFOX6)	33%	7.4 month	12.9 months	FGFR targeting might be reasonable for a small cohort of gastric cancer patients. Validation studies need to be performed.	[15]
	CTx (modified FOLFOX6) with Bemarituzumab	47%	9.5 months, *p* = 0.073	Not reached, *p* = 0.027		

Abbreviations: G: gastric; GEJ: gastroesophageal junction; ORR: objective response rate; mOS: median overall survival; mPFS: median progression free survival; FDA: Food and Drug Administration; EMA: European Medicines Agency; CTx: chemotherapy; ECX: Capecitabine plus Oxaliplatin and Epirubicin.

## Data Availability

Not applicable.
